# Synchronous neuroendocrine tumor and signet ring cell carcinoma in the stomach: a case report and review of literature

**DOI:** 10.3389/fmed.2025.1561231

**Published:** 2025-05-08

**Authors:** Jian Han, Ying Wang, Hanlin Mu, Jingmei Liu

**Affiliations:** ^1^Department of Gastroenterology, Tongji Hospital of Tongji Medical College, Huazhong University of Science and Technology, Wuhan, China; ^2^Department of Pathology, Tongji Hospital of Tongji Medical College, Huazhong University of Science and Technology, Wuhan, China; ^3^Department of Radiology, Tongji Hospital of Tongji Medical College, Huazhong University of Science and Technology, Wuhan, China

**Keywords:** synchronous gastric neoplasms, signet ring cell carcinoma, neuroendocrine tumor, gastric adenocarcinoma, gastric cancer simulating submucosal tumor

## Abstract

Synchronous neoplasms of the stomach are uncommon. Here we presented an unusual case with coexistence of neuroendocrine tumor and signet ring cell carcinoma in the stomach. Gastroscopic examination of a 66-year-old male patient showed a submucosal tumor-like mass with an ulcer on the surface located in the anterior wall of the lower segment of gastric body, confirmed by subsequent biopsy as a signet ring cell carcinoma. Moreover, we also found a slightly yellowish 6-mm protrusion simulating a polyp located in the lesser curvature of the upper segment of gastric body, and biopsy revealed it was a neuroendocrine tumor. The patient underwent a total gastrectomy with a standard D2 lymph node dissection. Pathohistological results led to the final diagnosis of synchronous neoplasms in the stomach, including a signet ring cell carcinoma and a neuroendocrine tumor.

## Introduction

Gastric cancer is the fifth most prevalent and third most fatal neoplastic disease globally (1). In China, the latest data from the National Cancer Center demonstrate that its morbidity and mortality rank the second and the third respectively, which seriously endangers public health (2, 3). Gastric signet ring cell carcinoma is a special histological type of gastric cancer, in which more than 50% of the isolated tumor cells contain intracellular mucin (4). Gastric neuroendocrine tumor is a rare type of gastric neoplasm arising from enterochromaffin-like cells of the gastric mucosa (5). The clinical presentation of gastric neuroendocrine tumors is not specific, and they are usually small-sized and multifocal, which could be missed or mis-diagnosed as polyps during gastroscopy. Most of the time, gastric neuroendocrine tumors are diagnosed by biopsy (5). The coexistence of gastric signet ring cell carcinoma and gastric neuroendocrine tumor in the same stomach is very rare (6, 7). We report this case and discuss the potential etiologies and pathological features of this rare presentation.

## Materials and methods

Immunohistochemistry for E-cadherin (20874-1-AP, 1:5000, Proteintech), MLH1 (clone OTI1C1, 1:150, ZSGB-BIO), PMS2 (clone OTI2G5, 1:150, ZSGB-BIO), MSH2 (15520-1-AP, 1:200, Proteintech), MSH6 (clone OTI5D1, 1:150, ZSGB-BIO), HER2 (60311-1-Ig, 1:1600, Proteintech), CD56 (clone OTI1A3, 1:150, ZSGB-BIO), synaptophysin (clone OTI1D4, 1:150, ZSGB-BIO), chromogranin A (clone OTI2F2, 1:500, ZSGB-BIO), SSTR2 (20404-1-AP, 1,200, Proteintech) and Ki-67 (clone OTI3D11, 1:50, ZSGB-BIO) were performed using a Leica Automated Staining System.

## Case description

**Figure 1:**
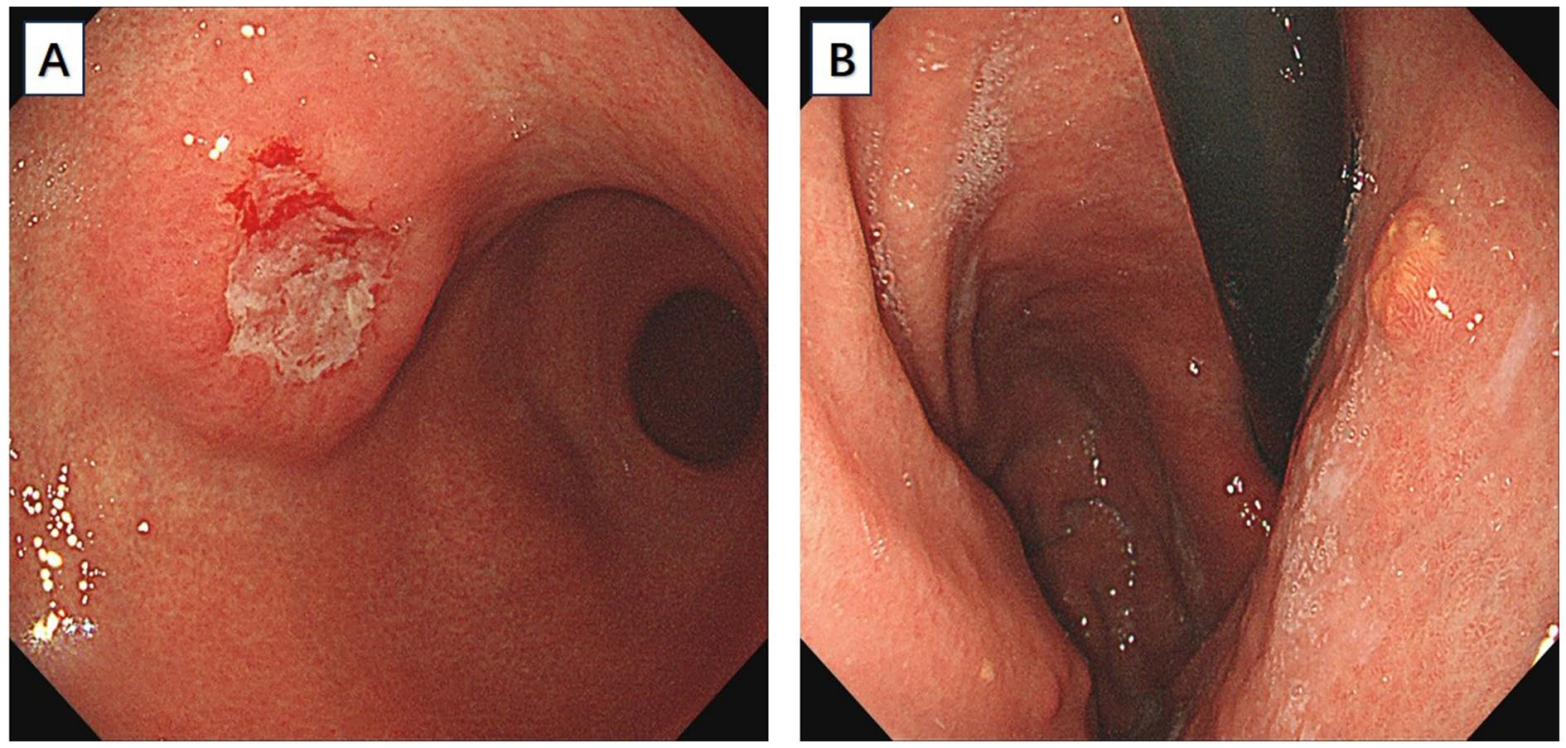
**(A)** An ulcerated mass morphologically similar to submucosal tumor was located at the anterior wall of the lower segment of gastric body. **(B)** A slightly yellowish 6-mm protrusion was located at the lesser curvature of the upper segment of gastric body.

**Figure 2:**
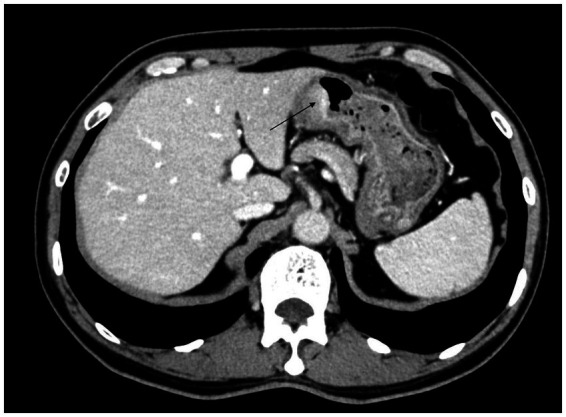
Abdominal computed tomography scan with intravenous contrast showed that the wall of gastric body was unevenly thickened and enhanced.

**Figure 3:**
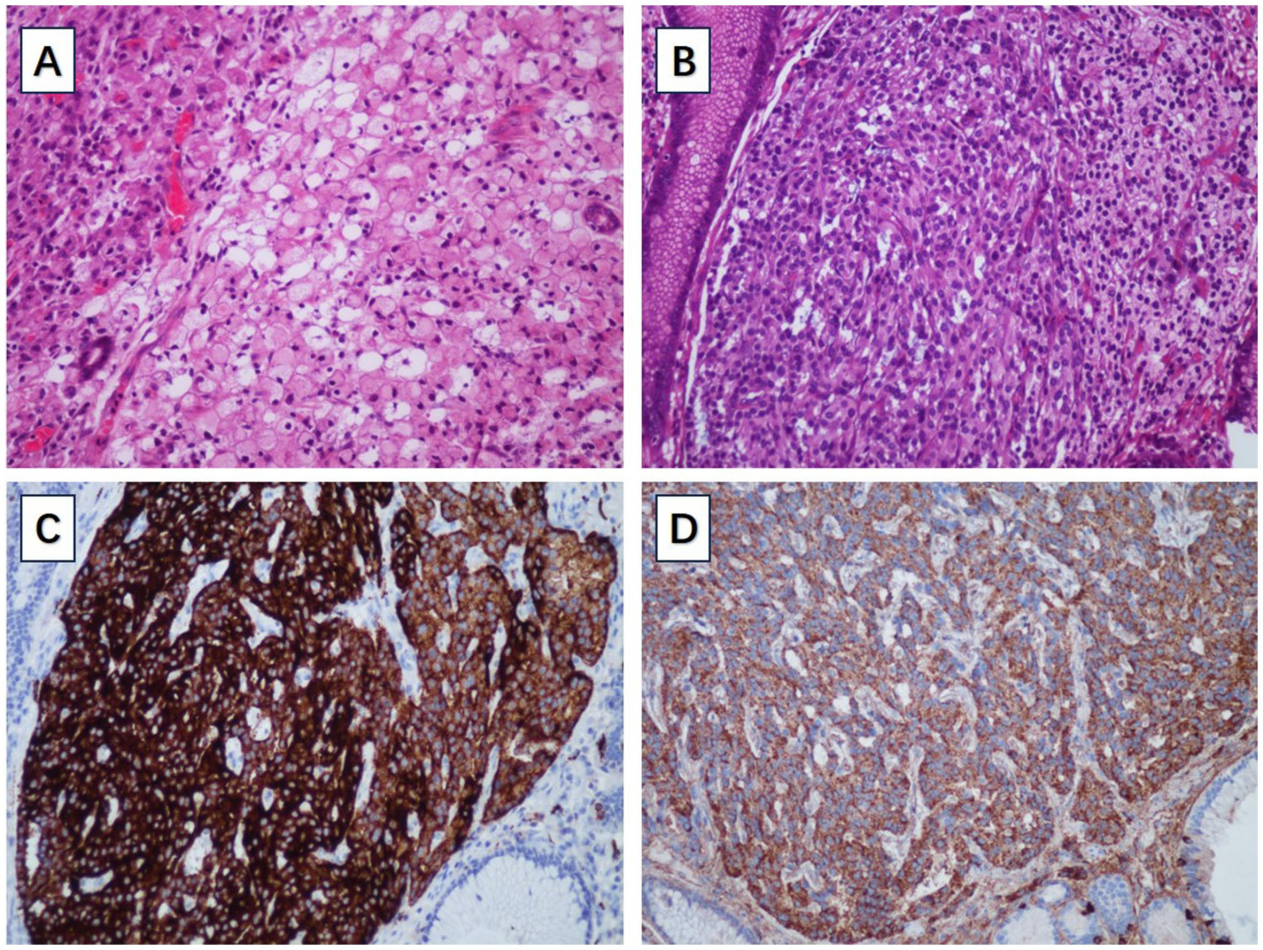
**(A)** The pathological examination of signet ring cell carcinoma. **(B)** The pathological examination of gastric NET. **(C)** The gastric NET was positive for synaptophysin. **(D)** The gastric NET was positive for chromogranin A.

A 66-year-old male patient presented to the local hospital with upper abdominal discomfort. He had no previous history of malignancy or other diseases. Blood routine test and blood biochemistry test were normal. Tumor marker tests showed that the level of carcinoembryonic antigen (CEA), carbohydrate antigen 19–9 (CA19-9) and carbohydrate antigen 72–4 (CA72-4) were also normal. Upper gastrointestinal endoscopy showed an ulcerated mass in the gastric body. In order to confirm the diagnosis, the patient came to our hospital. Reexamination of gastroscopy showed that a submucosal tumor-like protrusion with an ulcerated lesion was located at the anterior wall of the lower segment of gastric body (Figure 1A), as the endoscopists found in the local hospital, and biopsy was performed. Furthermore, we also found a slightly yellowish protrusion with a size of 0.6 cm located at the lesser curvature of the upper segment of gastric body (Figure 1B), and biopsy of the protrusion was also taken for pathological examination. The biopsy results of the ulcerated mass confirmed the diagnosis of signet ring cell carcinoma, and the biopsy results of the 0.6-cm protrusion confirmed the diagnosis of neuroendocrine tumor graded as G1 with Ki-67 positivity (1% of labeled cells in the hot-spots). Abdominal computed tomography scan with intravenous contrast showed that the wall of gastric body was unevenly thickened and enhanced and the serous membrane was smooth, without increased and enlarged adjacent regional lymph nodes (Figure 2). Thereafter, the patient underwent a total gastrectomy with a standard D2 lymph node dissection. During the surgery, 17 lymph nodes, including 13 lesser curvature lymph nodes and 4 greater curvature lymph nodes, were removed. The histopathological findings of the surgical specimen confirmed the diagnosis of synchronous signet ring cell carcinoma and neuroendocrine tumor, with a background mucosa of chronic atrophic gastritis. The tumor cells of the signet ring cell carcinoma invaded into the muscularis propria without adjacent regional lymph nodes involvement, staging T2N0M0, which were positive for E-cadherin, MLH1, PMS2, MSH2, MSH6 and Ki-67 (80% of labeled cells in the hot-spots), indicating microsatellite-stable, and negative for HER2. The neuroendocrine tumor graded as G1 was located in the lamina propria without neural invasion and vessel invasion. Immunostaining indicated that the NET cells were positive for CD56, synaptophysin, chromogranin A, SSTR2 and Ki-67 (1% of labeled cells in the hot-spots) (Figures 3A–D). No histological continuity was found between the signet ring cell carcinoma and the neuroendocrine tumor. Postoperatively, the patient recovered uneventfully and had no surgical complications. According to the final staging as T2N0M0, we recommended the patient closely regular follow-up rather than receiving additional adjuvant treatment. Given that often in these circumstances clinical pictures of sarcopenia up to cachexia occur (8), we recommended the patient to visit the department of clinical nutrition regularly for potential nutritional problems.

## Discussion

Gastric cancer is a deadly disease with poor overall survival statistics throughout the world (9). Upper gastrointestinal endoscopy has become an important tool in the diagnosis of patients with gastric neoplasms. However, it is reported that up to 6.7% of gastric neoplasms may be missed during gastroscopic examination (10). It is worth noting that an increasing number of synchronous gastric neoplasms are being discovered. To find out the important blind spots in screening gastroscopic examination, researchers retrospectively analyzed data about missed synchronous gastric neoplasms and concluded that the characteristics of the missed lesions included small size, upper one-third and middle one-third location, posterior wall location and flat or superficial appearance as compared with detected lesions (11, 12). Our present case is a case of almost missed synchronous gastric neoplasms including a submucosal tumor-like signet ring cell carcinoma and a very small neuroendocrine tumor.

Gastric carcinoma showing features of a submucosal tumor is uncommon. The prevalence of gastric carcinoma resembling submucosal tumor has been reported to be 0.2–0.62% (13). According to the World Health Organization (WHO) classification, gastric signet ring cell carcinoma, an unfavorable subtype of gastric adenocarcinoma, belongs to the type of poorly cohesive carcinoma (4, 14, 15). Gastric signet ring cell carcinomas are more prevalent among young women, with carcinomas mostly located in the middle one-third and lower one-third of the stomach. As is known to all, gastric signet ring cell carcinomas in early stage often present as whitish flat superficial lesions without gross mucosal abnormality such as ulcer and obvious protrusion or depression (16). However, gastric signet ring cell carcinomas may present as submucosal tumor-like lesions with progression to advanced stage or submucosal invasion (17–19), which have higher degrees of malignancy with a higher risk of lymph node metastasis and distant metastasis (14). In our present case, the gastric submucosal tumor-like lesion was pathologically confirmed by surgical resection to be an advanced stage signet ring cell carcinoma invading into the muscularis propria layer. Therefore, when a gastric submucosal tumor-like lesion is encountered during gastroscopic examination in clinical practice, we should consider the possibility of gastric carcinoma and carefully differentiate it from other subepithelial lesions.

Gastric neuroendocrine tumors are neoplasms arising from enterochromaffin-like (ECL) cells of the gastric oxyntic mucosa, comprising 1.9–2.2% of all the neuroendocrine tumors and 5–15% of all the gastro-entero-pancreatic neuroendocrine tumors. Typical neuroendocrine tumors of the stomach often present as submucosal protruding lesions, with a yellowish or reddish appearance under white light gastroscopy, sometimes with a central depression, and sometimes may be missed or misdiagnosed as polyps (20).

Synchronous multiple gastric neoplasm is a special type of gastric neoplasm with at least two neoplasms identified at different locations of the stomach without anatomical and pathological continuity (21). The phenomenon that signet ring cell carcinoma and neoplasm of another type synchronously occur in the same stomach is rare. Several case reports have described this condition (6, 7, 22–24), and the clinicopathological features of the previously reported cases and the present case are listed in Table 1.

**Table 1:** Reported cases of synchronous signet ring cell carcinoma and other tumor in the stomach.

Reference	Age (years)	Gender	Signet ring cell carcinoma		Another tumor		Treatment
			Location	Depth	Location	Pathology	
Our present case	66	Male	Body	Muscularis propria	Body	NET	Total gastrectomy
6	63	Male	Body	Intramucosal	Body	NET	Distal gastrectomy
7	55	Female	Antrum-body junction	Lamina propria	Antrum-body junction	NET	Distal gastrectomy
22	58	Male	Body	Muscularis propria	Body	GIST	Neoadjuvant imatinib plus total gastrectomy plus chemotherapy
23	82	Male	Body	Serosa	Body	GIST	Palliative wedge resection
24	56	Male	Body and antrum	Serosa	Body and antrum	MALT	Total gastrectomy plus chemotherapy

In the reported cases of synchronous neoplasms of signet ring cell carcinoma and neoplasm of another type in the stomach, all of the gastric signet ring cell carcinomas were located in the middle one-third or lower one-third of the stomach, and in our case, the signet ring cell carcinoma is located in the lower segment of gastric body, which is consistent with the viewpoint that gastric signet ring cell carcinoma is mostly located in the middle one-third and lower one-third of the stomach (14). So far, only two case reports described synchronous signet ring cell carcinoma and neuroendocrine tumor in the stomach (6, 7). In the first case, multiple nodules of 0.2–0.8 cm in diameter were detected in the anterior wall near the greater curvature of gastric body during gastroscopic examination, and mucosal biopsy was taken. With the help of immunohistochemistry, the histopathological finding of the specimen confirmed the diagnosis of multiple neuroendocrine tumors with positive expression of neuron-specific enolase, synaptophysin and chromogranin A. Subsequently, the patient received distal gastrectomy. Surprisingly, a superficially flat lesion with a size of 3.0 cm × 2.0 cm which was missed during preoperative gastroscopic examination was identified beside the lesser curvature of gastric body and was confirmed as an intramucosal early-stage signet ring cell carcinoma. The carcinoma cells contained mixed mucin, positive for both Alcin blue and Periodic Acid Schiff reaction, but were negative for the neuroendocrine markers. Because of the superficially flat appearance, this intramucosal early-stage signet ring cell carcinoma was missed during preoperative gastroscopic examination. In the second case, a submucosal nodule with a size of 1.0 cm at the posterior wall of antrum-body junction near the greater curvature was detected during gastroscopic examination. Biopsy was taken and histopathological findings led to the diagnosis of neuroendocrine tumor. Two weeks later, the patient received distal gastrectomy. Surprisingly, a signet ring cell carcinoma which was failed to be detected during preoperative gastroscopic examination was identified at 2.0 cm distal to the neuroendocrine tumor after systematic inspection and adequate dissection. Unlike the first case, this signet ring cell carcinoma was tiny with a size of 0.5 cm and thus was easy to be missed during preoperative gastroscopic examination. Our present case is the third case of synchronous signet ring cell carcinoma and neuroendocrine tumor in the stomach. However, different from the previous two cases, we have already identified the existence of two neoplasms of different pathological types during preoperative gastroscopic examination. Firstly, we noticed the large submucosal tumor-like signet ring cell carcinoma. Secondly, through a meticulous preoperative gastroscopic examination in the entire stomach, we also discovered a tiny neuroendocrine tumor which is very easy to be missed.

Therefore, we should perform gastroscopic examination meticulously every time. Even if a neoplasm was found during previous gastroscopic examination, we should be alert to the existence of synchronous gastric neoplasms that might otherwise be missed, especially those lesions with small size, upper one-third and middle one-third location, posterior wall location or flat/superficial type. There is currently no clear rules or regulations on whether preoperative gastroscopy is necessary, but comprehensive gastroscopy screening is very meaningful.

Nevertheless, considering the mechanism of tumorigenesis, it is not exactly elucidated whether the synchronicity of signet ring cell carcinoma and neuroendocrine tumor is incidental or there is a causative factor inducing the development of tumors of different histological types in the same organ. Gastric neuroendocrine tumor and gastric carcinoma share common risk factors including atrophic gastritis, hypoacidity and hypergastrinemia (25). As is known to all, autoimmune gastritis is associated with both type 1 gastric neuroendocrine tumor and gastric carcinoma (26). In 2015, a study described the occurrence of gastric carcinoma in autoimmune gastritis patients with type 1 gastric neuroendocrine tumor in a retrospective case series. In 4 (23.5%)/17 type 1 gastric neuroendocrine tumor patients, gastric adenocarcinoma occurred, including 3 cases of intestinal-type adenocarcinoma and 1 case of signet ring cell carcinoma (27). In 2019, Kubo K et al. reported a case of synchronous gastric adenocarcinoma and type 1 neuroendocrine tumor associated with autoimmune gastritis, and the two lesions were en bloc resected in one attempt by endoscopic submucosal dissection with negative margins. These lesions were pathologically diagnosed as neuroendocrine tumor graded as G1 and intramucosal well-differentiated tubular adenocarcinoma (28). These cases show that gastric carcinoma may frequently occur in patients with type 1 gastric neuroendocrine tumor. In 2007, Schott M et al. reported a case with multiple endocrine neoplasia (MEN) type 1 with gastrinoma and multiple type 2 gastric neuroendocrine tumors, and the patient additionally developed gastric signet ring cell carcinoma. Immunohistochemical studies showed diminished E-cadherin expression of gastric neuroendocrine tumors in comparison to normal gastric mucosa and showed loss of E-cadherin expression in the signet ring cells. This case suggested that gastrinoma/type 2 gastric neuroendocrine tumor-associated hypergastrinemia led to diminished E-cadherin expression and promoted carcinogenesis of signet ring cell carcinoma (29), which could also explain the frequent occurrence of carcinoma in patients with type 1 gastric neuroendocrine tumor. Pathologists, oncologists and surgeons should be aware of this interesting condition, and further research may be needed.

## Data Availability

The original contributions presented in the study are included in the article/Supplementary material, further inquiries can be directed to the corresponding author.
